# Non-communicable diseases in Brazil: a flood of data is coming! Doenças não-transmissíveis: uma inundação de dados está chegando!

**DOI:** 10.1590/1516-3180.2015.13340806

**Published:** 2015-08-03

**Authors:** Paulo Andrade Lotufo

**Affiliations:** I MD, DrPH. Full Professor, Department of Internal Medicine, Faculdade de Medicina da Universidade de São Paulo (FMUSP), São Paulo, Brazil.

## FIRST DATA-GENERATION PERIOD

Over the last two or three decades, academic researchers within health sciences frequently complained about the “drought” of information concerning chronic diseases in Brazil. This comment, by qualified physicians and scientists, was made much more frequently than would be justified by the reality of epidemiological production relating to non-communicable diseases, albeit restricted to mortality data and surveys. Even though both mortality data and surveys present relatively limited scope for reaching conclusions, the data produced were enough to understand some aspects of the epidemiological profile of chronic diseases in Brazil.

The mortality data was sufficiently accurate to show that a decline in cardiovascular diseases was occurring in Brazil, in contrast with other countries with the same level of economic development.^1^ In addition, survey data made it possible to ascertain the following points: (1) premature heart disease rates in Brazil were higher than in affluent countries;^2^ (2) cancer mortality among Japanese descendants in São Paulo showed differences according to the generation of migration, compared with individuals living in Japan;^3^ (3) Brazil had the highest death rate due to stroke in the Western world;^4^ (4) the burden of cardiovascular diseases was inversely associated with formal education levels among Brazilian municipalities;^5^ (5) the decline in the risk of death due to heart disease was not taking place uniformly, such that the pace was slower among people living in the poorest neighborhoods, compared with the wealthiest ones in São Paulo;^6^ and (6) the impact of the smoking habit on all causes of death in Brazil.^7^

Likewise, surveys addressing diabetes have been extremely useful for planning diabetes control programs over the whole country, among adults, pregnant women and the indigenous population.^8^^,^^9^^,^^10^ Every year since 2006, VIGITEL (Surveillance System of Risk and Protective Factors for Chronic Non-Communicable Diseases through Telephone Interviews), which is a telephone-based behavioral survey conducted among the 27 state capitals of Brazil, has been providing data on dietary habits, obesity, alcohol intake, physical activity and smoking habit.^11^ However, the most important effort by the Ministry of Health and the academic community has been the National Health Survey (“Pesquisa Nacional de Saúde”).^12^ The concept, design and preliminary results of this survey are presented in this issue of the *Journal.*

## SECOND DATA-GENERATION PERIOD

Since the beginning of this century, the academic and government sectors have been making joint efforts to provide better information through new studies: longitudinal studies, hospital registry studies and randomized trials. The stage of maturation of these studies is not uniform, but they are leading to increased levels of publication, at a fast rate. Table 1 summarizes some observational studies addressing non-communicable diseases that were designed and funded in Brazil.^13^^,^^14^^,^^15^^,^^16^^,^^17^^,^^18^^,^^19^^,^^20^

**Table 1.**
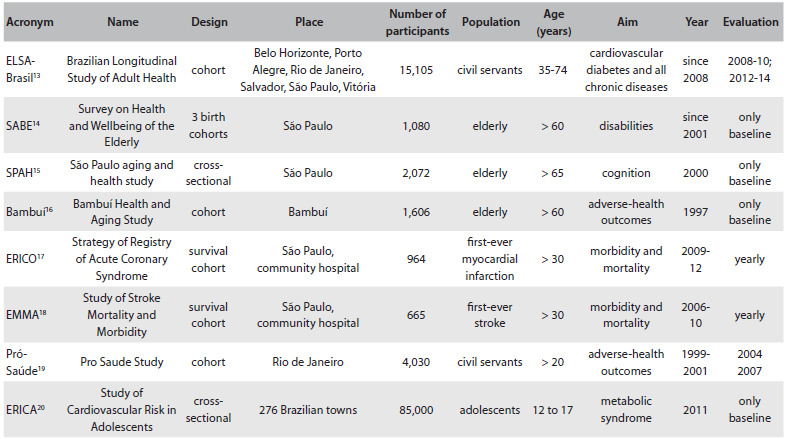
Observational studies in Brazil addressing the epidemiology of chronic diseases

Indeed, these new studies with much more data will open up a new window with impacts on National Health System policies, professional activity, the industry and science on the bench. Over the next issues of the Journal, each of these studies will be presented in greater detail.

Concluding, the drought of epidemiological information has come to an end in Brazil. Now, we will need to prepare ourselves so that we do not drown in the flood of data that is coming.
